# Does Ownership Matter? An Overview of Systematic Reviews of the Performance of Private For-Profit, Private Not-For-Profit and Public Healthcare Providers

**DOI:** 10.1371/journal.pone.0093456

**Published:** 2014-12-01

**Authors:** Cristian A. Herrera, Gabriel Rada, Lucy Kuhn-Barrientos, Ximena Barrios

**Affiliations:** 1 Department of Public Health, Faculty of Medicine, Pontificia Universidad Católica de Chile, Santiago, Chile; 2 Evidence Based Health Care Program, Faculty of Medicine, Pontificia Universidad Católica de Chile, Santiago, Chile; 3 Department of Internal Medicine, Faculty of Medicine, Pontificia Universidad Católica de Chile, Santiago, Chile; 4 National Health Technology Assessment Commission, Ministry of Health Chile, Santiago, Chile; RAND Corporation, United States of America

## Abstract

**Introduction:**

Ownership of healthcare providers has been considered as one factor that might influence their health and healthcare related performance. The aim of this article was to provide an overview of what is known about the effects on economic, administrative and health related outcomes of different types of ownership of healthcare providers -namely public, private non-for-profit (PNFP) and private for-profit (PFP)- based on the findings of systematic reviews (SR).

**Methods and Findings:**

An overview of systematic reviews was performed. Different databases were searched in order to select SRs according to an explicit comprehensive criterion. Included SRs were assessed to determine their methodological quality. Of the 5918 references reviewed, fifteen SR were included, but six of them were rated as having major limitations, so they weren't incorporated in the analyses. According to the nine analyzed SR, ownership does seem to have an effect on health and healthcare related outcomes. In the comparison of PFP and PNFP providers, significant differences in terms of mortality of patients and payments to facilities have been found, both being higher in PFP facilities. In terms of quality and economic indicators such as efficiency, there are no concluding results. When comparing PNFP and public providers, as well as for PFP and public providers, no clear differences were found.

**Conclusion:**

PFP providers seem to have worst results than their PNFP counterparts, but there are still important evidence gaps in the literature that needs to be covered, including the comparison between public and both PFP and PNFP providers. More research is needed in low and middle income countries to understand the impact on and development of healthcare delivery systems.

## Introduction

Several strategies exist to deliver healthcare to the population within a country. In terms of the providers, they can present, in general, 3 types of ownership: public, private non-for-profit (PNFP) and private for-profit (PFP). In public models, government owns and administers health care delivery institutions, with mostly public funding. On the other hand, PFP corporations are owned by shareholders or investors. They usually distribute some proportion of surplus or profits (net revenues less expenses) to owners, and have the purpose of increasing the wealth of shareholders within the boundaries of law. Lastly, PNFP institutions either do not have owners or are owned by “members” (religious organizations, communities, non-governmental organizations, regional health authorities or hospital boards). They cannot distribute surplus to those who control the organization, and its purpose is to fulfill a stated mission (provide healthcare, teaching, research, etc.) maintaining economic sustainability to do so [1]. Whether a provider is organized in any of these ways may affect structure, process, and outcome determinants of quality of care and health indicators [2] such as quality and appropriateness of care, efficiency/productivity, access to care, equity, cost containment, etc. [3]

From a theoretical point of view, there are several reasons, factors and mechanisms to expect these three types of ownership to have different impacts in health and health care related performance [3]. It can be said that PFP companies result in greater efficiency and better results when profit maximization is its overriding activity, there are no barriers to entry in the market, and there is an observable and measurable outcome [1] [4] [5]. However, this is not necessarily true for the healthcare industry. More than profits, other objectives struggle to be in frontline such as patient welfare, prestige, research, teaching, among others; barriers of entry are abundant (e.g. technology, high capital investment requirements, regulations such as certification and accreditation); and outcomes are hardly measurable for consumers (patients), so the information asymmetry is an important market failure. In addition, PFP providers may have fewer resources to spend on care because of taxes and their over-emphasized cost control in order to achieve the highest return on investment possible. Consequently, this can result in less qualified staff and/or less investment on equipment or technology [6]. PNFP providers face a different scenario, because they don't pursue the primary goal of making profits, which change the incentives for managers and clinicians (therefore, the organizational behavior). Usually, these organizations don't pay taxes, and reinvest the entire surplus (if existing) in improving the service [1] [7] [8]. Regarding the problems with PNFP providers, they are supposed to lack the incentive of making profits. Also, coordination across the healthcare system might be more difficult to achieve, for example, to tackle epidemics or to implement guidelines or interventions in an equal way throughout a region or country. This is also a problem of PFP providers. Finally, public providers, having a social welfare goal, are considered to generate a more equitable and coordinated system. However, it is argued that they have to deal with administrative bureaucracy and are often dependent on the budget and policies that political forces within a country or region may decide [6].

There are factors independent of ownership that may also affect the performance of providers [9]. Who pays for care and the extent to which it is paid for out of pocket or by a third party may interact with the impacts of health care provision under different types of ownership. Additionally, factors such as the characteristics of patients health status; differences in the amount of resources spent on the types of ownership being compared; the level of income of the country (low-, middle- or high-income); the mechanisms/mediators such as staffing, profit margins and tax rates; and corruption, can be considered that might influence the performance of health care providers. It is also relevant to point out that PNFP and PFP ownership can be local, national, international or single independent facilities.

In practice, the three types of ownership in healthcare provision exist in most countries with different proportion of each of them. The debate about who owns and administers the institutions providing care has been intense in high income countries such as USA and Canada [10] [11], but it is present worldwide. For example, on the supply side, in Canada and the USA there are mostly PNFP healthcare institutions, and in the UK has a largely public delivery system. Another example can be found in Chile, where the provision of healthcare is mixed, and the number of healthcare institutions with more than 10 hospitalization beds in the private sector reaches 184 centers while in the public sector there are 196. The latter accounts for 70% of hospitalization beds in the country [12]. In many low and middle income countries, private providers play a major role in the delivery system [13] [14] [15] [16], mainly because the public sector is not always sufficiently well-equipped and financed to provide high quality health services that are accessible to all citizens [17]. Hanson and Berman [18], based on a sample of low and middle-income countries, found that nearly 40% of doctors practice privately and 24% of available hospital beds are private (includes for-profit and non-profit). They also found that in Asia private providers offer nearly 26% of all beds available, compared to 33% in Africa. On the demand side, Saksena et al. 2012 [19] found that more than half of the utilization of health care services was at public facilities in 27 of 39 low income countries, and for 18 of them, was more than 80% of services. In South Asia, about three quarters of children from the poorest income quintile with acute respiratory diseases that seek health care go to a private provider [20], and across 26 African countries about forty five percent of sick children from the poorest income quintile go to a formal or informal private provider [21].

As it can be predicted, a large amount of different arrangements can be found under the term “ownership of healthcare providers”, and therefore, many systematic reviews can be –and has been- conducted regarding different questions about specific types and combinations of interventions; outcomes; conditions, problems or populations; and/or adverse effects of it. For this reason, we decided to conduct an “overview of systematic reviews” (OSR). The Cochrane Collaboration Handbook defines it as “reviews designed to compile evidence from multiple SRs of interventions into one accessible and usable document” [22]. An OSR is an ideal design to put together the existing body of evidence related with the ownership of health care providers and it can help to generate new hypothesis for future research and to guide policy decisions. In this context, the aim of this article is to provide an overview of what is known about the effects of different types of ownership of healthcare providers based on the findings of SR.

## Methods

We built upon the methodology for elaborating OSR provided in the Cochrane Collaboration Handbook [23] and the publication of Smith V, et al [24]. We didn't previously published the protocol of the overview. Supporting PRISMA checklist is available as supporting information; see Checklist S1.

### Criteria for considering reviews for inclusion

#### Type of participants

Any type of healthcare providers, such as primary care centers, hospitals, nursing homes, specialized medical centers, etc. The studies may have been conducted in any country.

#### Type of interventions

We included SR of studies evaluating the compared effects of the different types of ownership of healthcare providers: public, PNFP and/or PFP. Regarding the reviews that presented the results of private providers without separating between PNFP and PFP, we decided to include them but pointing out the limitations of this approach.

#### Type of outcomes

We included reviews reporting any of the following outcomes:

- Health related outcomes (e.g. mortality, morbidity, infections rates, preventive activities, etc.),
- Economic implications (e.g. payments, costs, debt, efficiency, etc.), or
- Management results (e.g. quality indicators, employees satisfaction, patient satisfaction, medical errors, length of stay, etc.).

#### Methodological characteristics

In order to include a review all of the following criteria were fulfilled:

1. Some explicit criteria for inclusion of studies are mentioned.
2. A list of included studies is provided. If a list is not provided we intended to extract it from tables or text. If a review fulfills all criteria but this, we contacted the authors by email.
3. Included studies have quantitative analyses, such as randomized controlled trials (RCTs), controlled clinical trials (CCTs), controlled before and after studies (CBAs), interrupted time series (ITSs), observational studies, among others.
4. Presents the results of studies in an individual basis, whether as numerical data or as a summary table or text.

The reviews must be published from the year 2002 onwards and no language restrictions were considered in their selection.

### Search methods for identification of reviews

We searched the following databases from the year 2002 till May 2013:

- Medline (www.pubmed.org). Search strategy can be found in the Appendix S1.
- Database of Abstracts of Reviews of Effects (http://www.crd.york.ac.uk/CMS2Web/AboutDare.asp)
- Health Technology Assessment Database (http://www.crd.york.ac.uk/CMS2Web/AboutHTA.asp)
- Health Systems Evidence Database (http://www.healthsystemsevidence.org/)
- Epistemonikos – PDQ-Evidence Database (http://www.epistemonikos.org/http://www.pdq-evidence.org)
- Google Scholar

We checked the reference lists of the included SR and contacted the main authors of them.

### Data collection and analysis

#### Selection of reviews

wo independent authors screened the titles and abstracts retrieved in the databases search to identify reviews that appeared to meet the inclusion criteria. To the articles selected, two independent authors applied the selection criteria to the full text of each one of them to select the finally included SR. Disagreements were resolved by consensus.

#### Data extraction and management

Two independent authors used a standardised form to extract data, including: complete citation, stated aim of the review, details of the search (databases and search dates), number and design of studies, participant healthcare providers, type of ownership and comparisons, factors considered for adjustment in the included studies of the review and countries where the studies were conducted. For the outcomes we extracted the number of studies favouring any type of ownership or no effect, and/or any quantitative data reported.

#### Assessment of methodological quality of included reviews

Two independent authors assessed the reliability of SRs that met the inclusion criteria using the checklist developed by the SUPPORT and SURE collaborations (Appendix S2). In this way, each review was categorised as:

- Reliable (only minor limitations)
- Important limitations (limitations that are important enough that it would be worthwhile to interpret the results of this review cautiously)
- Major limitations (having limitations that are important enough that the results of the review are not reliable and they should not be included in the overview)

We excluded from the analyses the reviews that were scored as having major limitations.

#### Data synthesis

We reported individual review narrative summaries of their results, and organized review data according to the type of ownership and their comparison(s) being investigated.

## Results

**Figure 1:**
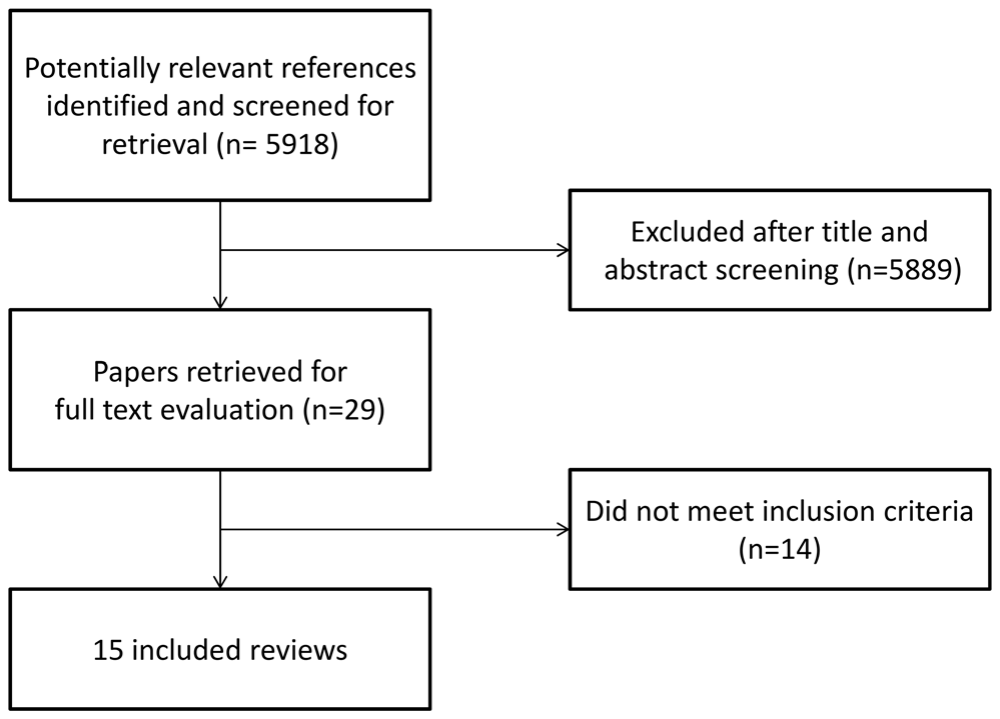
Search strategy results and description of studies selection process.

The initial search yielded a total of 5918 articles, of which 29 were estimated to be potentially eligible. After the revision of full texts, we included 15 SR. Appendix S3 provides a list of the excluded studies and the reasons for exclusion. Figure 1 shows the process of the selection of reviews. The scope of the included SR is presented in Table 1.

**Table 1:** Scope of the included systematic reviews.

Review	Aim	Last search	N° of included studies	Countries of primary studies
Basu 2012	To evaluate available data on public and private sector performance across the key domains of health systems competencies.	Aug 2011	102	LMIC
Berendes 2011	To systematically identify and summarise the results of studies that directly compare the quality of private providers and public services in relation to ambulatory health care in LMICs.	Dec 2010	80	LMIC
Comondore 2009	To examine the quality of care in for-profit and not-for-profit (privately and publicly owned) nursing homes to enhance the evidence base for public policy.	April 2006	82	HIC
Devereaux 2002	To determine whether a difference in adjusted mortality rates exists between hemodialysis patients receiving care in private for-profit vs private not-for profit dialysis centers.	2002	8	HIC
Devereaux 2002	To explore the relative effect of private for-profit versus private not for-profit delivery of hospital care on patient mortality.	2002	15	HIC
Devereaux 2004	To study the payments for patient care received at private for-profit compared with private not-for profit hospitals.	2002	8	HIC
Eggleston 2008	To examine what factors explain the diversity of findings regarding hospital ownership and quality of care.	July 2004	31	HIC
Hillmer 2005	To perform a systematic review examining the association between the profit status of North American nursing homes and the quality of care.	October 2002	38	HIC
Mogyorósy 2004	To review the literature of studies comparing hospital financial performance and the quality of care before and after conversion from public or non-profit status to for-profit in the USA.	2004	Not clear	HIC
Montagu 2011	To answer the question: what difference exist in health outcomes following treatment in public or private settings in low- and middle income countries?	2010	21	LMIC
Rosenau 2003	To study the performance of for-profit and nonprofit inpatient psychiatric health care providers.	2002	17	HIC
Rosenau 2003	To assess the performance differences between private for-profit and private nonprofit U.S. health care providers published since 1980.	2002	149	HIC
Shen 2007	To examine what factors explain the diversity of findings regarding hospital ownership and financial performance.	July 2004	40	HIC
Sibbel 2012	To analyse the current state of research on the question of whether private hospitals are more efficient, or whether the public ones are just plain worse operators of clinics.	2009	8	HIC
Tiemann 2012	To perform a review on the findings of empirical research on the association between hospital ownership and efficiency in Germany.	July 2011	20	HIC

LMIC: low and middle income countries; HIC: high income countries.

The methodological quality appraisal resulted in one reliable SR, eight with important limitations, and the remaining six were rated as having major limitations [25] [26] [27] [28] [29] [30]. Therefore, we only considered nine SRs for presenting, analyzing and discussing their results. In the following section we present their findings separated by the 3 possible comparisons -PNFP vs PFP, PNFP vs Public, and PFP vs Public- and mixed combinations of ownership types. Table 2 shows a summary of the results of the analyzed SR.

**Table 2:** Summary of the results in the analyzed systematic reviews.

Review	Healthcare providers type	Type of ownership compared	Adjustment factors	Outcomes		
				Health	Economic	Managerial
Berendes et al. 2011	Ambulatory centers	Public vs PNFP-PFP*	None mentioned.	-	-	The formal private sector was better for drug availability, responsiveness, and effort. Overall, the median differences were modest and not statistically significant.
Comondore et al. 2009	Nursing homes	Public and PNFP vs PFP	Age; Severity of illness (comorbidities); Presence or absence or severity of dementia; Payment status of residents (government funded vs privately funded).	-	-	PNFP nursing homes deliver higher quality care than do PFP nursing homes. “Quality of care” was most commonly measured as the number of staff per resident or level of training of staff, physical restraints, pressure ulcers, and regulatory (government surveys) deficiencies.
Devereaux et al. 2002	Hemodyalisis centers	PNFP vs PFP	Age, race, and cause of end-stage renal disease. Also, income, education, number of years receiving dialysis, market share of the dialysis facility (ie, competition), and whether the dialysis facility was part of a multinational chain corporation. 2 studies had over-adjustments because of the inclusion of staffing land skill levels.	Hemodialysis care in PNFP centers is associated with a lower risk of mortality compared with care in PFP.	-	-
Devereaux et al. 2002	Hospitals	PNFP vs PFP	Patients' severity of illness and socioeconomic status, hospital teaching status.	PFP hospitals were associated with an increased risk of death compared to PNFP.	-	-
Devereaux et al. 2004	Hospitals	PNFP vs PFP	Age, sex, education, ethnicity, marital status, income, community living, number of activities of daily living, cognitive awareness, bladder/bowel control, comorbidity, primary diagnosis at index admission, market characteristics, year of index admission, number of hospital beds and hospital teaching status. Two studies were unadjusted.	-	PFP hospitals were associated with higher payments for care compared to PNFP.	-
Eggleston et al. 2008	Hospitals	Public vs PNFP/PNFP vs PFP	Patients' case mix and demographics, hospital-level and market-level control variables.	Whether PFP and public hospitals have higher mortality rates or rates of adverse events than their PNFP counterparts depends on a study's data source as well as time period and region covered.	-	-
Montagu et al. 2011	Primary, secondary and terciary levels of care	PFP and PNFP vs Public	Comorbidities, socioeconomic status.	Very low quality evidence shows that patients in private healthcare settings are less likely to die than patients in a public healthcare setting. Moderate quality evidence says that unsuccessfully completed tuberculosis treatment is higher in private than public healthcare settings.	-	-
Sibbel et al. 2012	Acute care hospitals	PFP vs PNFP/PFP vs Public	None mentioned	-	Because of weaknesses and a variety of differences in the methodological structure of the studies a really convincing answer whether PFP hospitals are more efficient cannot be derived from the results.	-
Shen et al. 2007	Hospitals	Public vs PNFP/PNFP vs PFP	Patients' case mix and demographics, hospital-level and market-level control variables.	-	Studies that control for a wider range of counfounding factors, and have transformed health care costs into logarithm when used as the dependent find statistically significant less differences between PNFP and PFP hospitals. No differences were found between public and PNFP hospitals.	-

PNFP: private non-for-profit; PFP: private for-profit.

* Berendes et al. 2011 performed sub-group analyses with Sub-Saharian countries where they did separate between PFP and PNFP, but this was a secondary exploration. The main analysis in the paper was with this 2 types of ownership together.

### PNFP versus PFP

Six SR addressed this comparison. All the included studies were from HIC and have observational designs. Devereaux 2002 [31] performed a meta-analysis of 14 studies that adjusted for confounding factors finding that PFP hospitals were associated with an increased risk of death (relative risk 1.020, 95% CI 1.003–1.038; p = 0.02) compared with PNFP hospitals in the USA. One study of perinatal mortality that evaluated 1.642.002 patients in 243 hospitals and adjusted for potential confounders also demonstrated an increased risk of death in PFP hospitals (RR 1.095, 95% CI 1.050–1.141; p<0.0001). Devereaux 2002 [32] carried on a meta-analysis with the 8 included studies demonstrating that PFP dialysis facilities were associated with an increased risk of death (RR 1.09; 95% CI, 1.05–1.12; p<0.001; P = 0.004 for heterogeneity. Over 0.05, heterogeneity can be considered to be low), and this was supported in all sensitivity analyses including the one with the 3 well-adjusted and un-confounded studies (RR 1.09; p<0.001; P = 0.5 for heterogeneity). Regarding economic outcomes, Devereaux 2004 [33] in their primary meta-analysis demonstrated that PFP hospitals were associated with higher payments for care (relative payments for care 1.19; 95% CI 1.07–1.33, p = 0.001, I2 for heterogeneity  = 90%. Over 50%, heterogeneity can be considered to be high). Specialty versus general hospitals was the only analysis that significantly explained heterogeneity, and the pooled estimates from both the 3 studies that evaluated specialty hospitals and the 5 studies that evaluated general hospitals showed higher payments for care at PFP hospitals (relative payments for care 1.48; 95% CI 1.15–1.89, and 1.11; 95% CI 1.00–1.23, respectively).

Two reviews explored the diversity of findings across studies. Shen et al. [6] and Eggleston et al. [34], taking articles only from the US, used an “effect size” measure for each included study (without combining them) and performed meta-regression analyses, through which they compared the impact of ownership regarding financial performance and quality of care in hospitals, respectively. In Eggleston et al. [34], of the 25 studies analyzing mortality, one favored PFP and one PNFP, and all the rest did not have significant tendencies. Of the 12 studies comparing adverse events, two showed higher rates in PFP and the others did not have significant tendencies. Among the factors that might explain the differences between the studies, they found that the estimates of the relationship between hospital ownership and adverse patient outcomes differ systematically mainly according to a study's data source (surveys representative of the US –i.e. National Long-term Care Survey- conclude that PFPs and PNFP hospitals do not statistically differ in measured outcomes, but only 14% of studies had this). In addition, the time period examined (ownership effects based on 1980s data suggest a wider quality gap between private hospitals), and region covered (if uses data that is representative of US hospitals, it finds higher mortality or adverse events rates in PFP hospitals) were considered as relevant by the authors but these features were statistically significant at 0.1 level of significance. Shen et al. [6] studied costs, revenues, profit margins, and efficiency in hospitals. 18 out of 40 studies analyzed cost differences. Of them, 9 showed higher costs in PFP, 4 lower costs in PFP, and 5 no statistically significant differences. Studies that control for a wider range of counfounding factors, and have transformed health care costs into logarithm when used as the dependent variable (health care costs are highly skewed) found statistically significant less differences. Among the 11 studies comparing hospital revenue, 6 showed significantly higher revenues in PFP hospitals and 5 did not found significant differences. For profits margins, 6 studies showed that PFP hospitals earns significantly higher profit than PNFP hospitals, and 8 found no significant differences. Regarding efficiency, 7 studies found that PFP hospitals are less efficient, 5 that are more efficient, and 2 found no significant differences. Finally, Sibbel et al. [35] studied efficiency in acute care hospitals of HICs using data envelopment analysis. The authors found 5 of 8 studies revealing that public and PNFP hospitals are more efficient than those in PFP ownership, one concluding the opposite, and 2 not demonstrating any significant differences between the different hospital ownerships. Due to methodological constraints, they concluded that it is not possible to ascertain that PFP hospitals are more efficient than PNFP or public ones.

### PNFP versus Public

Three SR explored this comparison. For LMIC, in the subgroup of African Sub-Saharian countries in Berendes et al. [36], they found a not statistically significant tendency in favor of PNFP ambulatory centers. Responsiveness and clinical practice (e.g. compliance with clinical guidelines) categories were significantly better in PNFP, and competence (e.g. knowledge tests or exams) better in public centers.

Regarding HIC, Shen et al. [6] found not much difference between both hospital ownership types. In the meta-regression analysis, they found that studies whose research focus is not ownership tend to find public hospitals significantly less costly than PNFP hospitals. In Eggleston et al. [34], of the 12 studies analyzing mortality, four showed higher mortality rates in public hospitals, and all the rest did not have significant tendencies. Of the 5 studies comparing adverse events, one showed higher rates in public hospitals and the others did not have significant tendencies. Among the factors that might explain the differences between the studies, they found that studies including Medicare claims are associated with findings of significantly higher mortality and adverse event rates in public hospitals.

### PFP versus Public

Only Berendes et al. [36] explored specifically this comparison. Analyzing a subgroup of African Sub-Saharian countries compared PFP with public ambulatory centers, finding a not statistically significant tendency in favor of PFP facilities. Drug availability and clinical practice categories were statistically significant better in PFP, but competence was better in public centers.

### Comparisons with combination of ownership types

Berendes et al. [36] and Montagu et al. [37] did not separate between PFP and PNFP providers in their main analyses. Berendes et al. [36] compared public ambulatory healthcare providers to PNFP and PFP providers together, studying their structural (i.e. buildings, equipment, drugs availability), delivery (i.e. patient satisfaction) and technical quality (i.e. competence of the staff). Structurally, no difference was found on buildings, equipment and material, but drugs availability was significantly higher in private providers. In terms of service delivery, private providers performed better in responsivenness, waiting times, and average consultation times. There was no difference on patient satisfaction. Technical quality (competence and clinical practice) was similar between both types of providers. Montagu et al. [37] performed a meta-analysis with 15 observational studies finding that patients in private healthcare settings are less likely to die than patients in a public healthcare setting (OR 0.60; 95% CI 0.41–0.88), with this evidence rated as been of very low quality. In the subgroup analyses, they found that inpatient setting, lower middle income countries and non-tuberculosis treatments were the areas where private providers were significantly better than public. Also, the pooled analysis showed that patients in a private healthcare facility are more likely to have unsuccessfully completed tuberculosis treatment than patients in a public healthcare facility (OR 2.04; 95% CI 1.07–3.89), rating this evidence as of moderate quality.

Comondore [2] analyzed together the data for public and PNFP, considering this as ‘not-for-profit nursing homes’, and comparing them to PFP. They performed four meta-analyzes, finding that not-for profit homes have more or higher quality staffing (ratio of effect 1.11; 95% CI 1.07–1.14), p<0.001, I2 = 91.6%), and a lower prevalence of pressure ulcers (OR 0.91; 95% CI 0.83–0.98, p = 0.02, I2 = 52.1%), with an absolute risk reduction of 0.59% (0.13% to 1.12%) and a relative risk reduction of 8.4% (1.9% to 16%). The use of physical restraints and the deficiencies in governmental regulatory assessments were both fewer in not-for profit homes, but with not statistically significant differences. Exploring heterogeneity, they separated public from PNFP nursing homes, and comparing the latter witn PFP homes the results stayed the same.

## Discussion

The overview found that few of the included SR had strong methodological quality, where only one of them was classified as reliable. According to the findings of the considered SRs, ownership does seem to have an effect on health and healthcare related outcomes. In the comparison of PFP and PNFP providers, the meta-analyses found significant differences in terms of mortality of patients and payments in hospitals of HIC, both being higher in PFP facilities. Studies that controlled for adequate confounding variables, in general, confirmed this tendency. In terms of quality indicators, differences were not that clear between these two types of ownerships. For PNFP and public providers, in both HIC and LMIC there were no statistically significant tendencies, showing that in the current stage of knowledge no major differences have been found between these 2 types of providers. Finally, in the few studies that compared PFP and public providers, no clear differences were found.

As there are theoretical and empirical reasons to expect that some of these 3 types of ownership might have different outcomes among them, results of primary studies and SRs that analyzed the data of any of this forms together (e.g. PFP and PNFP together as one type of ownership) must be interpreted very carefully as they might be misleading.

It is remarkable that after controlling for different factors that might explain or influence the performance of healthcare providers, in many cases ownership appears to have an effect, particularly, between PFP and PNFP. As theory predicted, many market failures acts to have a translation into the outcomes of PFP providers. For instance, the quality of equipments, staff qualification and number, overtreatment in some cases, etc. might be some practical consequences directed by the misaligned incentives that profit puts to these organizations. This can be an explanation for this finding and should be considered further.

### Implications for research

Four different areas can be commented regarding this point. First, primary research seem to be more abundant in HIC, so it becomes important to develop more studies in LMIC to understand how ownership of providers in different levels and types of them affects different outcomes in these nations. Second, primary or ambulatory care has not been explored in a SR in HIC, which may help to determine the effect of ownership on this level. Third, the comparisons of PNFP vs public and PFP vs public providers have been seldom explored with both primary studies and SR methodologies. And fourth, a more rigorous SR comparing efficiency outcomes (i.e. with data envelopment or stochastic frontier analysis research as primary studies) for healthcare providers (i.e. hospitals) would be an important advance to the health economics literature. As a general comment, many of currently published SRs will be soon not up to date.

In summary, there is a need of developing high quality primary studies, particularly, in LMIC. As it is difficult to carry on trials (i.e. randomized controlled trials) in this area, observational studies such as interrupted time series or controlled before-after studies could be preferably performed. Also, a rigorous updated SR, separating clearly between the 3 possible comparisons, would be a contribution to current knowledge in the matter.

### Implications for policy

Policymakers make decisions about governance arrangements within providers in healthcare systems. In this process, systematic reviews can assist them to make better-informed decisions [38]. Regarding the ownership of them, PFP facilities seem to have worst results on health and economic outcomes. If this type of ownership is mantained or decided to be promoted, these findings might be considered when thinking about regulation policies that could diminish or control these negative consequences. PNFP and public providers seem to be similar in terms of their results, so policymaking processes might want to look to other considerations when deciding about what ownership to prefer or promote.

In general terms, monitoring and evaluation efforts of changes in ownership of healthcare providers should always be considered in order to better understand the effect of one or other type of ownership.

## Conclusion

This is the first OSR about ownership of healthcare providers that explores with a broad perspective the effects of this governance characteristic. PFP providers seem to have worst results than their PNFP counterparts, but there are still important evidence gaps in the literature that needs to be covered. The results here presented would be useful for future research and for decision-making processes by policy makers.

## Supporting Information

Checklist S1 **PRISMA Checklist.**
(DOCX)

Appendix S1 **Medline search strategy.**
(DOCX)

Appendix S2 **Table: Quality appraisal of the included systematic reviews.**
(DOCX)

Appendix S3 **List of excluded studies.**
(DOCX)
