# Supplementary material for: Does Ownership Matter? An Overview of Systematic Reviews of the Performance of Private For-Profit, Private Not-For-Profit and Public Healthcare Providers
Source: PLoS One. 2014 Dec 1;9(12):e93456. doi: 10.1371/journal.pone.0093456 (PMC4249790; doi:10.1371/journal.pone.0093456)
Supplement: Appendix S1 — Medline search strategy. (DOCX) [file pone.0093456.s002.docx]

Appendix S2: Medline search strategy

1. meta analysis[Publication Type]

2. meta analysis[Title/Abstract]

3. meta analysis[MeSH Terms]

4. review[Publication Type]

5. search*[Title/Abstract])

6. #1 OR #2 OR #3 OR #4 OR #5

7. “Ownership”[Mesh]

8. “Organizations, Nonprofit”[Mesh]

9. “Health Facilities, Proprietary”[Mesh]

10. non-for-profit

11. non-profit

12. non for profit

13. non profit

14. for-profit

15. for profit

16. (commercial AND (profit OR purpose OR organization OR ownership))

17. (non commercial AND (profit OR purpose OR organization OR ownership))

18. non-commercial

19. lucrative

20. non lucrative

21. charitable

22. altruistic

23. philanthropic

24. ngo

25. nongovernment*

26. non government*

27. #7 OR #8 OR #9 OR #10 OR #11 OR #12 OR #13 OR #14 OR #15 OR #16 OR #17 OR #18 OR #19 OR #20 OR #21 OR #22 OR #23 OR #24 OR #25 OR #26

28. #6 AND #27

29. Limits: published in the last 10 years
