# Supplementary material for: Does Ownership Matter? An Overview of Systematic Reviews of the Performance of Private For-Profit, Private Not-For-Profit and Public Healthcare Providers
Source: PLoS One. 2014 Dec 1;9(12):e93456. doi: 10.1371/journal.pone.0093456 (PMC4249790; doi:10.1371/journal.pone.0093456)
Supplement: Appendix S2 — Table: Quality appraisal of the included systematic reviews. (DOCX) [file pone.0093456.s003.docx]

Appendix S3. Table: Quality appraisal of the included systematic reviews.

|  | Methods used to identify, include and critically appraise studies | | | | | | Methods used to analyse the findings | | | | | |  |  |  |
| --- | --- | --- | --- | --- | --- | --- | --- | --- | --- | --- | --- | --- | --- | --- | --- |
| Review | Were the criteria used for deciding which studies to include in the review reported? | Was the search for evidence reasonably comprehen-sive? | Is the review reasonably up-to-date? | Was bias in the selection of articles avoided? | Did the authors use appropriate criteria to assess the risk for bias in analysing the studies that are included? | OVERALL | Were the characteristics and results of the included studies reliably reported? | Were the methods used by the review authors to analyse the findings of the included studies reported? | Did the review describe the extent of heterogeneity? | Were the findings of the relevant studies combined (or not combined) appropriately relative to the primary question the review addresses and the available data? | Did the review examine the extent to which specific factors might explain differences in the results of the included studies? | OVERALL | REVIEW RELIABILITY |  |  |
| Basu et al. 2012 | Yes | C/P | Yes | C/P | No | ML | No | Yes | No | No | C/P | IL | ML |  |  |
| Berendes et al 2011 | Yes | Yes | Yes | Yes | C/P | IL | C/P | Yes | Yes | Yes | C/P | IL | IL |  |  |
| Comondore et al 2009 | Yes | Yes | Yes | Yes | No | IL | Yes | Yes | Yes | Yes | Yes | R | IL |  |  |
| Devereaux et al. 2002 | Yes | C/P | Yes | Yes | C/P | IL | Yes | Yes | Yes | Yes | Yes | R | IL |  |  |
| Devereaux et al. 2002 | Yes | C/P | Yes | Yes | C/P | IL | Yes | Yes | Yes | Yes | Yes | R | IL |  |  |
| Devereaux et al. 2004 | Yes | C/P | Yes | Yes | C/P | IL | Yes | Yes | Yes | Yes | Yes | R | IL |  |  |
| Eggleston 2008 | Yes | C/P | Yes | C/P | C/P | IL | Yes | Yes | Yes | Yes | Yes | R | IL |  |  |
| Hillmer 2005 | C/P | C/P | Yes | C/P | No | IL | C/P | Yes | C/P | No | Yes | ML | ML |  |  |
| Mogyorósy 2004 | C/P | C/P | Yes | Yes | No | ML | C/P | Yes | Yes | C/P | C/P | IL | ML |  |  |
| Montagu 2011 | Yes | Yes | Yes | Yes | Yes | R | Yes | Yes | Yes | Yes | Yes | R | R |  |  |
| Rosenau 2003 | C/P | C/P | C/P | C/P | No | ML | C/P | Yes | No | No | No | ML | ML |  |  |
| Rosenau 2003 | C/P | C/P | C/P | C/P | No | ML | C/P | Yes | No | No | No | ML | ML |  |  |
| Shen et al. 2007 | Yes | C/P | Yes | C/P | C/P | IL | Yes | Yes | Yes | Yes | Yes | R | IL |  |  |
| Sibbel et al. 2012 | C/P | C/P | Yes | C/P | C/P | IL | C/P | Yes | Yes | C/P | Yes | Yes | IL |  |  |
| Tiemann et al. 2012 | Yes | No | Yes | C/P | No | ML | C/P | No | No | No | C/P | ML | ML |  |  |

C/P: Can’t tell / Partially; R: Reliable; IL: Important limitations; ML: Major limitations
