# Supplementary material for: Does Ownership Matter? An Overview of Systematic Reviews of the Performance of Private For-Profit, Private Not-For-Profit and Public Healthcare Providers
Source: PLoS One. 2014 Dec 1;9(12):e93456. doi: 10.1371/journal.pone.0093456 (PMC4249790; doi:10.1371/journal.pone.0093456)
Supplement: Appendix S3 — List of excluded studies. (DOCX) [file pone.0093456.s004.docx]

Appendix S4: List of excluded studies

1. Bell CA, Duncan G, Saini B. (2011) Knowledge, attitudes and practices of private sector providers of tuberculosis care: a scoping review. Int J Tuberc Lung Dis 15(8):1005–1017.

It is centered in exploring the role and results of the private sector on TB interventions, but does not compare according to the type of ownership (non-for-profit or for-profit).

1. Braithwaite J, Travaglia JF, Corbett A. (2011) Can Questions of the Privatization and Corporatization, and the Autonomy and Accountability of Public Hospitals, Ever be Resolved? Health Care Anal; 19:133–153.

It focuses on privatization and corporatization processes, but not specifically in its effects.

1. Costa AM, Silva KS, Bonan C. (2011) Organizações Não Governamentais na área da Saúde da Criança – revisão da literatura. Cien Saude Colet;16(7):3181-96.

It is focused on NGOs role in child healthcare, but does not compare it to other types of ownership.

1. Egan, M., M. Petticrew, et al. (2007). ""Profits before people"? A systematic review of the health and safety impacts of privatising public utilities and industries in developed countries." J Epidemiol Community Health 61(10): 862-70.

It analyzes the health impacts of privatization of building, water, paper, cement, bus, rail, mining, electric and gas companies.

1. Eggleston, K., L. Ling, et al. (2008). "Health service delivery in China: a literature review." Health Econ 17(2): 149-65.

Narrative review.

1. Guyatt GH, Devereaux P, Lexchin J, Stone SB, Yalnizyan A, Himmelstein D, et al. A systematic review of studies comparing health outcomes in Canada and the United States. Open Med. 2007;1(1):e27-36. PubMed PMID: 20101287. Epub 2007/01/01. eng.

It compares results between Canada and the United States but not according to the ownership status of the health care providers.

1. Holden, C. (2005). "Privatization and trade in health services: a review of the evidence." Int J Health Serv 35(4): 675-89

Narrative review about trade opening in countries with different institutional characteristics and its effect on the levels of private provision of healthcare.

1. Holmes, J. S. (1996). "The effects of ownership and ownership change on nursing home industry costs (Brief record)." Health Services Research 31(3): 327-346

Observational study.

1. Hyde JK, Shortell SM. (2012) The structure and organization of local and state public health agencies in the U.S.: a systematic review. Am J Prev Med;42(5 Suppl 1):S29-41.

It only analyzes public health agencies, not private facilities or organizations.

1. Hyman, D. A. and W. M. Sage (2006). "Subsidizing health care providers through the tax code: status or conduct?" Health Aff (Millwood) 25(4): W312-5

Perspective article.

1. Lee, C. L., T. L. Liu, et al. (2002). "Cost and care quality between licensed nursing homes under different types of ownership (Brief record)." Journal of Nursing Research 10(2): 151-160.

Observational study.

1. Levin A, Kaddar M. Role of the private sector in the provision of immunization services in low- and middle-income countries. Health Policy and Planning 2011; 26: i4-i12.

Does not compare the performance of the different types of providers’ ownership.

1. Schlesinger M, Gray BH. (2006) How Nonprofits Matter In American Medicine, And What To Do About It. Health Affairs. 25(4):W287-W303.

Narrative review.

1. Tsai, A. G. and B. Kinosian (2003). "The association between profit levels and quality of care in California nursing homes." Med Care 41(12): 1315-7

Editorial.
